# Development and external validation of a faecal immunochemical test-based prediction model for colorectal cancer detection in symptomatic patients

**DOI:** 10.1186/s12916-016-0668-5

**Published:** 2016-08-31

**Authors:** Joaquín Cubiella, Pablo Vega, María Salve, Marta Díaz-Ondina, Maria Teresa Alves, Enrique Quintero, Victoria Álvarez-Sánchez, Fernando Fernández-Bañares, Jaume Boadas, Rafel Campo, Luis Bujanda, Joan Clofent, Ángel Ferrandez, Leyanira Torrealba, Virginia Piñol, Daniel Rodríguez-Alcalde, Vicent Hernández, Javier Fernández-Seara, on behalf of the COLONPREDICT study investigators, Joaquín Cubiella, Pablo Vega, María Salve, Marta Díaz-Ondina, Irene Blanco, Pedro Macía, Eloy Sánchez, Javier Fernández-Seara, María Teresa Alves, Enrique Quintero, Natalia González-López, Victoria Álvarez Sánchez, José Mera, Juan Turnes, Fernando Fernández-Bañares, Victoria Gonzalo, Mar Pujals, Josepa Ribes, Ramón Cleries, Xavier Sanz, Jaume Boadas, Sara Galter, Rafel Campo, Marta Pujol, Eva Martínez-Bauer, Antonio Alsius, Luis Bujanda, Jesús Bañales, María J. Perugorria, Joan Clofent, Ana Garayoa, Ángel Ferrández, Marina Solano Sánchez, Leyanira Torrealba, Virginia Piñol, Daniel Rodriguez-Alcalde, Jorge López-Vicente, Vicent Hernández, Felipe Iglesias

**Affiliations:** 1 Gastroenterology Department, Complexo Hospitalario Universitario de Ourense, Rua Ramón Puga 52-54, 32005 Ourense, Spain; 2 Instituto de Investigación Biomedica (IBI) Ourense, Pontevedra y Vigo, Vigo, Rua Ramón Puga 52-54, 32003 Ourense, Spain; 3 Clinical Analysis Department, Complexo Hospitalario Universitario de Ourense, Rua Ramón Puga 52-54, 32003 Ourense, Spain; 4 NECOM Group, University of Vigo, Campus Universitario As Lagoas, Marcosende, 36310 Vigo, Spain; 5 Gastroenterology Department, Hospital Universitario de Canarias, Instituto Universitario de Tecnologías Biomédicas (ITB) & Centro de Investigación Biomédica de Canarias (CIBICAN), Universidad de La Laguna, Carretera de Ofra, s/n, 38320 San Cristóbal de La Laguna, Santa Cruz de Tenerife Spain; 6 Gastroenterology Department, Complejo Hospitalario de Pontevedra, Av. Montecelo, 36164 Casas Novas, Pontevedra, Spain; 7 Gastroenterology Department, Hospital Universitari Mútua de Terrassa, Centro de Investigación Biomédica en Red de Enfermedades Hepáticas y Digestivas (CIBERehd), Plaça del Doctor Robert, 5, 08221 Terrassa, Barcelona Spain; 8 Gastroenterology Department, Consorci Sanitari de Terrassa, Carr. Torrebonica, S/N, 08227 Terrassa, Barcelona Spain; 9 Gastroenterology Department, Hospital de Sabadell, Corporació Sanitària i Universitària Parc Taulí, Parc Taulí, 1, 08208 Sabadell, Barcelona Spain; 10 Donostia Hospital, Biodonostia Institute, University of the Basque Country UPV/EHU, (CIBERehd), Paseo del Doctor Begiristain 117, 20080 San Sebastian, Guipuzcoa Spain; 11 Gastroenterology Department, Hospital de Sagunto, Avenida Ramón y Cajal, S/N, 46520 Sagunto, Valencia Spain; 12 Servicio de Aparato Digestivo, Hospital Clínico Universitario, IIS Aragón, University of Zaragoza, (CIBERehd), Avda. San Juan Bosco, 15, 50009 Zaragoza, Spain; 13 Gastroenterology Department, Hospital Dr. Josep Trueta, Avenida de Francia, s/n, 17007 Girona, Spain; 14 Digestive Disease Section, Hospital Universitario de Móstoles Río Júcar, s/n, 28935 Mostoles, Madrid Spain; 15 Gastroenterology Department, Vigo, Pontevedra, Spain

**Keywords:** Colorectal cancer, Faecal immunochemical test, Colonoscopy, Diagnostic accuracy, Risk stratification, Prompt diagnosis

## Abstract

**Background:**

Risk prediction models for colorectal cancer (CRC) detection in symptomatic patients based on available biomarkers may improve CRC diagnosis. Our aim was to develop, compare with the NICE referral criteria and externally validate a CRC prediction model, COLONPREDICT, based on clinical and laboratory variables.

**Methods:**

This prospective cross-sectional study included consecutive patients with gastrointestinal symptoms referred for colonoscopy between March 2012 and September 2013 in a derivation cohort and between March 2014 and March 2015 in a validation cohort. In the derivation cohort, we assessed symptoms and the NICE referral criteria, and determined levels of faecal haemoglobin and calprotectin, blood haemoglobin, and serum carcinoembryonic antigen before performing an anorectal examination and a colonoscopy. A multivariate logistic regression analysis was used to develop the model with diagnostic accuracy with CRC detection as the main outcome.

**Results:**

We included 1572 patients in the derivation cohort and 1481 in the validation cohorts, with a 13.6 % and 9.1 % CRC prevalence respectively. The final prediction model included 11 variables: age (years) (odds ratio [OR] 1.04, 95 % confidence interval [CI] 1.02–1.06), male gender (OR 2.2, 95 % CI 1.5–3.4), faecal haemoglobin ≥20 μg/g (OR 17.0, 95 % CI 10.0–28.6), blood haemoglobin <10 g/dL (OR 4.8, 95 % CI 2.2–10.3), blood haemoglobin 10–12 g/dL (OR 1.8, 95 % CI 1.1–3.0), carcinoembryonic antigen ≥3 ng/mL (OR 4.5, 95 % CI 3.0–6.8), acetylsalicylic acid treatment (OR 0.4, 95 % CI 0.2–0.7), previous colonoscopy (OR 0.1, 95 % CI 0.06–0.2), rectal mass (OR 14.8, 95 % CI 5.3–41.0), benign anorectal lesion (OR 0.3, 95 % CI 0.2–0.4), rectal bleeding (OR 2.2, 95 % CI 1.4–3.4) and change in bowel habit (OR 1.7, 95 % CI 1.1–2.5). The area under the curve (AUC) was 0.92 (95 % CI 0.91–0.94), higher than the NICE referral criteria (AUC 0.59, 95 % CI 0.55–0.63; *p* < 0.001). On the basis of the thresholds with 90 % (5.6) and 99 % (3.5) sensitivity, we divided the derivation cohort into three risk groups for CRC detection: high (30.9 % of the cohort, positive predictive value [PPV] 40.7 %, 95 % CI 36.7–45.9 %), intermediate (29.5 %, PPV 4.4 %, 95 % CI 2.8–6.8 %) and low (39.5 %, PPV 0.2 %, 95 % CI 0.0–1.1 %). The discriminatory ability was equivalent in the validation cohort (AUC 0.92, 95 % CI 0.90–0.94; *p* = 0.7).

**Conclusions:**

COLONPREDICT is a highly accurate prediction model for CRC detection.

**Electronic supplementary material:**

The online version of this article (doi:10.1186/s12916-016-0668-5) contains supplementary material, which is available to authorized users.

## Background

Colorectal cancer (CRC) is the most common tumour, the seventh cause of death and the fourth cause of years of life lost in Western Europe [1]. Health authorities have developed two strategies to reduce CRC-related impact: CRC screening and prompt diagnosis in symptomatic patients [2–6]. In order to reduce the delay between the onset of symptoms and diagnosis and improve prognosis, several criteria with high probability for CRC detection have been established. In this regard, the best known guidelines are the National Institute for Health and Care Excellence (NICE) criteria for suspected cancer [3]. Although patients meeting these criteria are more likely to have CRC, their specificity is low [7–9]. Moreover, these criteria are under the physician’s subjective evaluation [4].

In recent years, several CRC prediction models have been designed and validated in different settings [10]. Although diagnostic accuracy is acceptable and better than the existing referral criteria, these prediction models have not been widely implemented [11–13]. Nowadays, there are several potential biomarkers available that could be used to determine the risk of CRC detection in symptomatic patients. A faecal immunochemical test (FIT) has proven to be a useful diagnostic test both for CRC screening in asymptomatic individuals and for diagnosis in symptomatic patients [8, 14–18]. Semiquantitative FIT allows for quantification of faecal haemoglobin (f-Hb) concentration. There are several prediction models in asymptomatic individuals for CRC detection based on FIT [19]. However, no one has evaluated the effect of FIT together with other clinical parameters to determine the risk of CRC in symptomatic patients [7–10].

On the basis of the hypothesis that a predictive model for CRC diagnosis based on symptoms, biomarkers and demographical information could improve the diagnostic accuracy of the NICE referral criteria, we have carried out a cross-sectional study on symptomatic patients referred for colonoscopy to develop a CRC prediction model and have subsequently externally validated it in a different set of patients.

## Methods

### Design

COLONPREDICT is a multicentre, cross-sectional, blinded study of diagnostic tests. The study aimed to create and validate a CRC prediction index based on available biomarkers and clinical and demographic data.

### Population

The derivation cohort consisted of consecutive patients with gastrointestinal symptoms referred for colonoscopy from primary and secondary health care to Complexo Hospitalario Universitario de Ourense, Spain. Exclusion criteria were age under 18, pregnancy, asymptomatic individuals who were undergoing colonoscopy for CRC screening, patients with a previous history of colonic disease who underwent a surveillance colonoscopy, patients requiring hospital admission, patients whose symptoms had ceased within 3 months before evaluation, and patients who declined to participate after reading the informed consent form. The study was approved by the Clinical Research Ethics Committee of Galicia (Code 2011/038). Patients provided written informed consent.

### Interventions

The Colonoscopy Research Into Symptom Prediction questionnaire was used to record symptoms and demographic data. This had been translated into Spanish after receiving permission from the authors [20]. Nurses specifically trained in the assessment of gastrointestinal symptoms administered the questionnaire to the patients. They also collected administrative information and determined if patients met any of the NICE referral criteria for CRC detection: patients ≥40 years with rectal bleeding and a change of bowel habit persisting ≥6 weeks; patients ≥60 years with rectal bleeding persisting ≥6 weeks without a change in bowel habit and without anal symptoms; patients ≥60 years with a change in bowel habit persisting ≥6 weeks without rectal bleeding; patients presenting a right lower abdominal mass consistent with involvement of the large bowel; patients presenting with a palpable rectal mass; or patients with unexplained iron deficiency anaemia (<11 g/100 mL in men, <10 g/100 mL in non–menstruating women) [3].

All individuals collected a faeces sample from one bowel movement without specific diet or medication restrictions the week before the colonoscopy. They were specifically instructed to sample a stool where no blood was visible. f-Hb concentration was assessed using the automated OC-SENSOR™ (Eiken Chemical Co., Tokyo, Japan) and faecal calprotectin was determined using a commercial ELISA kit (Bühlmann fCAL ELISA calprotectin, Bühlmann Laboratories AG, Basel, Switzerland). The stool sample for the f-Hb determination was collected using the OC-SENSOR probe. The stool sample for the faecal calprotectin determination was collected independently. We determined blood haemoglobin (b-Hb) and mean corpuscular volume with a Beckman Coulter Autoanalyzer (Beckman Coulter Inc., CA, USA) and serum carcinoembryonic antigen (CEA) using a chemiluminescent microparticle immunoassay (UniCel DXI 800; Beckman Coulter).

### Colonoscopy

Colonoscopy was performed blind for the questionnaire and analytical results. Before the colonoscopy, endoscopists performed a digital rectal examination as well as an anoscopy to determine anorectal findings. Bowel cleansing and sedation was performed as previously described [21]. We considered colonoscopy complete if caecal intubation was achieved. All colonoscopies were performed by experienced endoscopists (>200 colonoscopies per year). Endoscopists described all colorectal lesions and obtained biopsies if appropriate.

### Main outcome

The main outcome was CRC. We determined the location of CRC as rectum, distal or proximal to splenic flexure. Tumour staging was performed according to the American Joint Committee on Cancer (AJCC) classification 7th edition [22]. The secondary outcomes were advanced neoplasia (AN) and significant colonic lesion (SCL). We defined AN as CRC or advanced adenoma (≥10 mm, villous histology, high-grade dysplasia). SCL was defined as CRC, advanced adenoma, polyposis (>10 polyps of any histology, including serrated lesions), histologically confirmed colitis (any aetiology), polyps ≥10 mm, complicated diverticular disease (diverticulitis, bleeding), colonic ulcer and bleeding angiodysplasia. The remaining lesions were considered non-significant colonic lesions. Data from each individual were registered in an online database.

### Sample size calculation

The sample size for the derivation cohort was calculated on the hypothesis that our prediction index sensitivity for CRC detection would be better than the NICE referral criteria. Assuming that CRC prevalence was between 5 and 10 %, NICE referral criteria sensitivity for CRC was 80 % and our prediction index sensitivity for CRC was 90 %, a sample size of 2526 patients would provide 80 % power at a 5 % significance level using a two-sided test. [10] Assuming 10 % losses we would need a final sample size of 2778 patients. An interim analysis was performed after including 800 patients [8]. In this intermediate analysis, CRC prevalence was 12 % and the number of losses was 5 %. On the basis of these data, the final sample size required to include in the derivation cohort was 1607 patients.

### Development of the prediction model

Initially we performed a descriptive analysis where continuous variables were expressed as median [minimum–maximum] and qualitative variables as frequency and percentage. We determined potential associations between CRC and the independent variables with parametric/nonparametric tests (Chi-square, Student’s *t* test, Mann–Whitney). We studied correlations by exploratory data to detect a relationship or interaction between the different variables. Before logistic regression, we performed a univariate analysis using generalised additive models with smoothing splines for continuous variables. The objective of this analysis was to determine, in those non-linear variables, the different strata or classes. We introduced significant variables in this first analysis and those that could be of clinical interest in the multivariate logistic regression analysis (we eliminated those with colinearity or linear combination of others). We used the regression coefficients to construct a CRC prediction score, where the dependent variable was presence/absence of CRC. We calculated the R2 (a measure of variation) of the model for CRC detection and the area under the curve (AUC) in the receiver operating characteristic (ROC) curve. Finally, we also assessed the Akaike Information Criterion (AIC) and the Bayesian Information Criterion (BIC). The final model was chosen on the basis of the highest discriminatory ability measured with the AUC.

In order to evaluate the diagnostic yield of the final prediction model, we established two example thresholds with a 90 and 99 % sensitivity for CRC detection, and we determined the diagnostic accuracy for CRC and SCL at each threshold. According to these thresholds, we divided the cohort into three groups: high (values over the 90 % threshold), intermediate (values between the 90 and 99 % threshold) and low risk (values below the 99 % threshold) for CRC detection. We calculated the number of patients, the positive predictive value (PPV) and the number needed to endoscopy to detect a CRC and an SCL in each group. We compared our predictive model with the NICE referral criteria in two ways: (1) AUC using the Chi-square test of homogeneity of areas and (2) comparison of the sensitivity and specificity at the sensitivity thresholds established with the McNemar’s test. We additionally calculated the diagnostic accuracy in two additional example thresholds: 50 % sensitivity and 90 % specificity for CRC detection.

### External validation of the prediction model

The validation cohort included a prospective cohort of patients with gastrointestinal symptoms referred for colonoscopy in 11 hospitals in Spain. We collected the variables included in the model prospectively and we used the coefficients to calculate the COLONPREDICT score for each patient in the validation dataset. We also determined those patients that met the criteria for 90 and 99 % sensitivity. We compared the discriminatory ability of the model in the derivation and the validation cohorts with ROC curves and AUC on one side, and with the Chi-square test to determine differences in sensitivity and specificity at the established thresholds between both cohorts for CRC, AN and SCL detection.

### Diagnostic accuracy according to healthcare level

Finally, we performed a post hoc analysis of our model to determine if its diagnostic accuracy was modified on the basis of the healthcare level referring the patient for colonoscopy: primary versus secondary healthcare. In order to perform this analysis we grouped derivation and validation cohorts and we compared discriminatory ability with ROC curves, AUC, and sensitivity and specificity with the Chi-square test.

We report differences with 95 % confidence intervals (95 % CI). A *p*-value <0.05 was considered to be statistically significant. Analysis was carried out using SPSS statistical software, version 15.0 (SPSS Inc., Chicago, IL, USA) and EPIDAT 3.1 (Dirección Xeral de Saúde Pública, Santiago de Compostela, Spain).

## Results

### Description of the derivation cohort

**Fig. 1:**
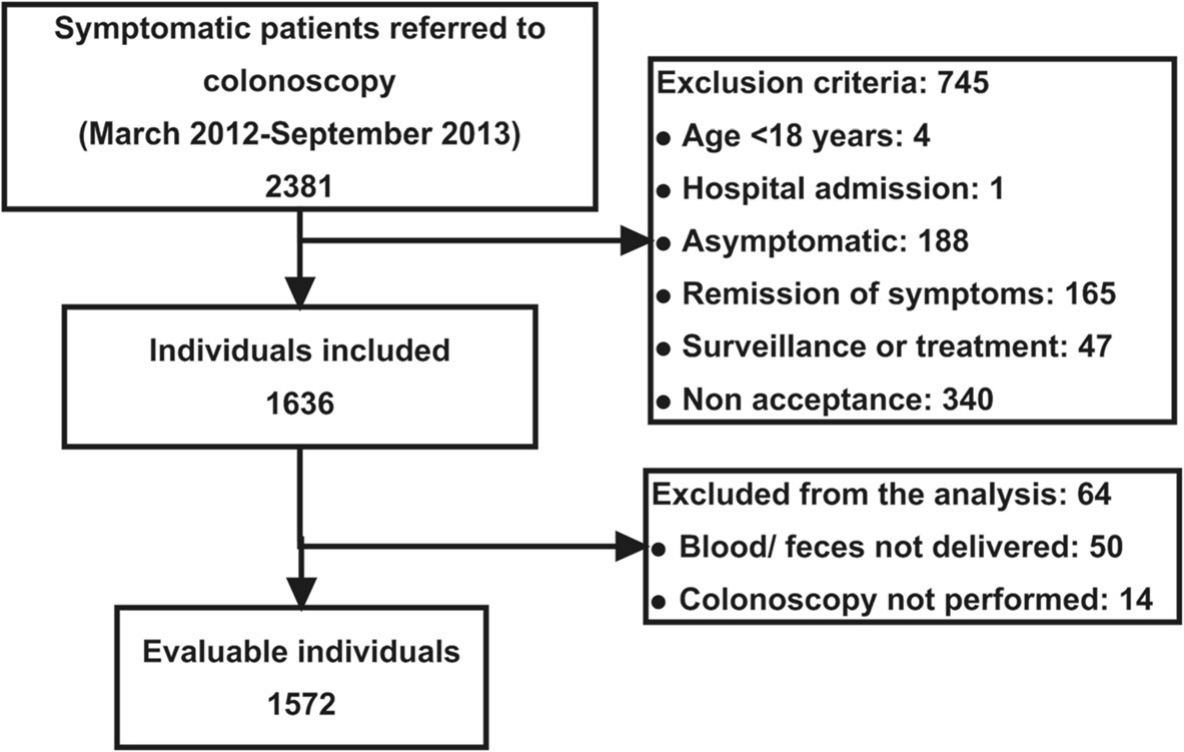
Enrolment of the patients included in the COLONPREDICT derivation cohort

Between March 2012 and September 2013, 2381 patients were referred for colonoscopy for the evaluation of symptoms. After excluding 745 patients due to exclusion criteria, 1636 patients were included in the initial cohort. Finally, 64 patients did not complete the study protocol, so there were 1572 evaluable patients (Fig. 1). We show the baseline characteristics of the patients included in Table 1. We detected CRC in 214 (13.6 %) patients, located in the rectum (37.4 %) and colon (43.5 % of total CRC distal and 19.2 % proximal to the splenic flexure). Tumour staging was 0 (2.8 %), I (18.6 %), II (25.1 %), III (37.7 %) and IV (15.8 %). Additionally, we found advanced adenomas in 251 patients (16.0 %), a polyp ≥10 mm with non-adenoma histology in 6 patients (0.4 %), colitis in 36 patients (2.3 %) and other SCLs in 6 patients (0.4 %). Overall, we detected a SCL in 463 patients (29.5 %).

**Table 1:** Baseline characteristics of the individuals included in the derivation cohort

Characteristics	Overall (*n* = 1572)	CRC (*n* = 214)	No CRC (*n* = 1358)	Significance
Age (years)^a^	68 (20–96)	74 (39–92)	68 (20–96)	<0.001
Sex (male)	810 (51.5 %)	138 (64.5 %)	672 (49.5 %)	1.8 (1.3–2.5)
Primary healthcare referral (yes)	360 (22.9 %)	67 (31.3 %)	293 (21.6 %)	1.6 (1.2–2.3)
NICE referral criteria (yes)	821 (52.2 %)	146 (68.2 %)	675 (49.7 %)	2.2 (1.6–2.9)
Symptoms				
• Abdominal pain (yes)	688 (43.8 %)	75 (35.0 %)	613 (45.1 %)	0.7 (0.5–0.9)
• Anal pain (yes)	360 (22.9 %)	39 (18.2 %)	321 (23.6 %)	0.7 (0.5–1)
• Change in bowel habit (yes)	899 (57.2 %)	132 (61.7 %)	767 (56.5 %)	1.2 (0.9–1.7)
• Rectal bleeding (yes)	942 (59.9 %)	141 (65.9 %)	801 (59.0 %)	1.3 (1–1.8)
• Incomplete evacuation (yes)	510 (32.4 %)	69 (32.2 %)	441 (32.5 %)	1 (0.7–1.3)
• Mucus on faeces (yes)	178 (11.3 %)	26 (12.1 %)	152 (11.2 %)	1.1 (0.7–1.7)
• Asthenia (yes)	671 (42.7 %)	106 (49.5 %)	565 (41.6 %)	1.4 (1–1.8)
• Weight loss (yes)	385 (24.5 %)	57 (26.6 %)	328 (24.2 %)	1.1 (0.8–1.6)
Duration of symptoms				
• <1 month	97 (5.5 %)	12 (5.6 %)	75 (5.5 %)	1.6 (0.8–3.1)
• 1–12 months	803 (51.1 %)	140 (65.4 %)	663 (48.8 %)	2.1 (1.5–2.9)
• >12 months	682 (43.4 %)	62 (29.0 %)	620 (45.7 %)	1
Previous colorectal diseases				
• Diverticulitis (yes)	64 (4.1 %)	6 (2.8 %)	58 (4.3 %)	0.6 (0.3–1.5)
• Irritable bowel syndrome (yes)	32 (2.0 %)	1 (0.5 %)	31 (2.3 %)	0.2 (0.02–1.5)
• Polyps (yes)	110 (7.0 %)	5 (2.3 %)	105 (7.7 %)	0.3 (0.1–0.7)
Colonoscopy in the last 10 years (yes)	314 (20.0 %)	10 (4.7 %)	304 (22.4 %)	0.2 (0.1–0.3)
FDRs with CRC (yes)	301 (19.1 %)	21 (9.8 %)	280 (20.6 %)	0.4 (0.3–0.7)
Treatment				
• Aspirin (more than one year)	224 (14.2 %)	25 (11.7 %)	199 (14.7 %)	0.7 (0.5–1.2)
• NSAIDs (yes)	263 (16.7 %)	27 (12.6 %)	236 (17.4 %)	0.7 (0.4–1.1)
• Laxative treatment (yes)	21 (1.3 %)	1 (0.5 %)	20 (1.5 %)	0.3 (0.1–2.3)
• Acenocoumarol (yes)	124 (7.9 %)	17 (7.9 %)	107 (7.9 %)	1 (0.6–1.7)
• Clopidogrel (yes)	60 (3.8 %)	10 (4.7 %)	50 (3.7 %)	1.3 (0.6–2.6)
Anorectal examination findings				
• Rectal mass (yes)	38 (2.4 %)	31 (14.5 %)	7 (0.5 %)	32.6 (14.2–75.3)
• Benign anorectal lesion (yes)	646 (41.1 %)	32 (15.0 %)	614 (45.2 %)	0.2 (0.1–0.3)
Laboratory results				
• Faecal haemoglobin (μg/g)^b^	5.8 (0.0–5902)	270 (0.0–1974)	5.8 (0.0–5902)	<0.001
• Faecal calprotectin (ng/mL)^b^	41.0 (0.0–5100)	120 (13.0–1072)	37.0 (0.0–5100)	<0.001
• Blood haemoglobin (g/dL)^b^	13.5 (6.3–19.1)	12.9 (7.2–17.8)	13.6 (6.3–19.1)	<0.001
• Mean corpuscular volume (fL)^b^	90.6 (56.7–118.7)	89.1 (62.7–110.5)	90.8 (56.7–118.7)	<0.001
• Serum CEA (ng/mL)^b^	1.6 (0.0–3701)	3.2 (0.1–2684)	1.5 (0.0–3701)	<0.001

Qualitative variables are expressed as absolute numbers and percentages. Quantitative variables are expressed as median and range

Differences were analysed with the Chi-square and the Cochran–Mantel–Haenszel statistics and expressed as the odds ratio and its 95 % confidence interval in the qualitative variables; and the ^a^Student’s *t* and ^b^Mann–Whitney U test in quantitative variables. Differences with *p* < 0.05 were considered statistically significant

*CEA* Carcinoembryonic antigen, *CRC* Colorectal cancer, *FDR* First-degree relative, *NSAIDs* Nonsteroidal anti-inflammatory drugs

### Development of the prediction model

**Fig. 2:**
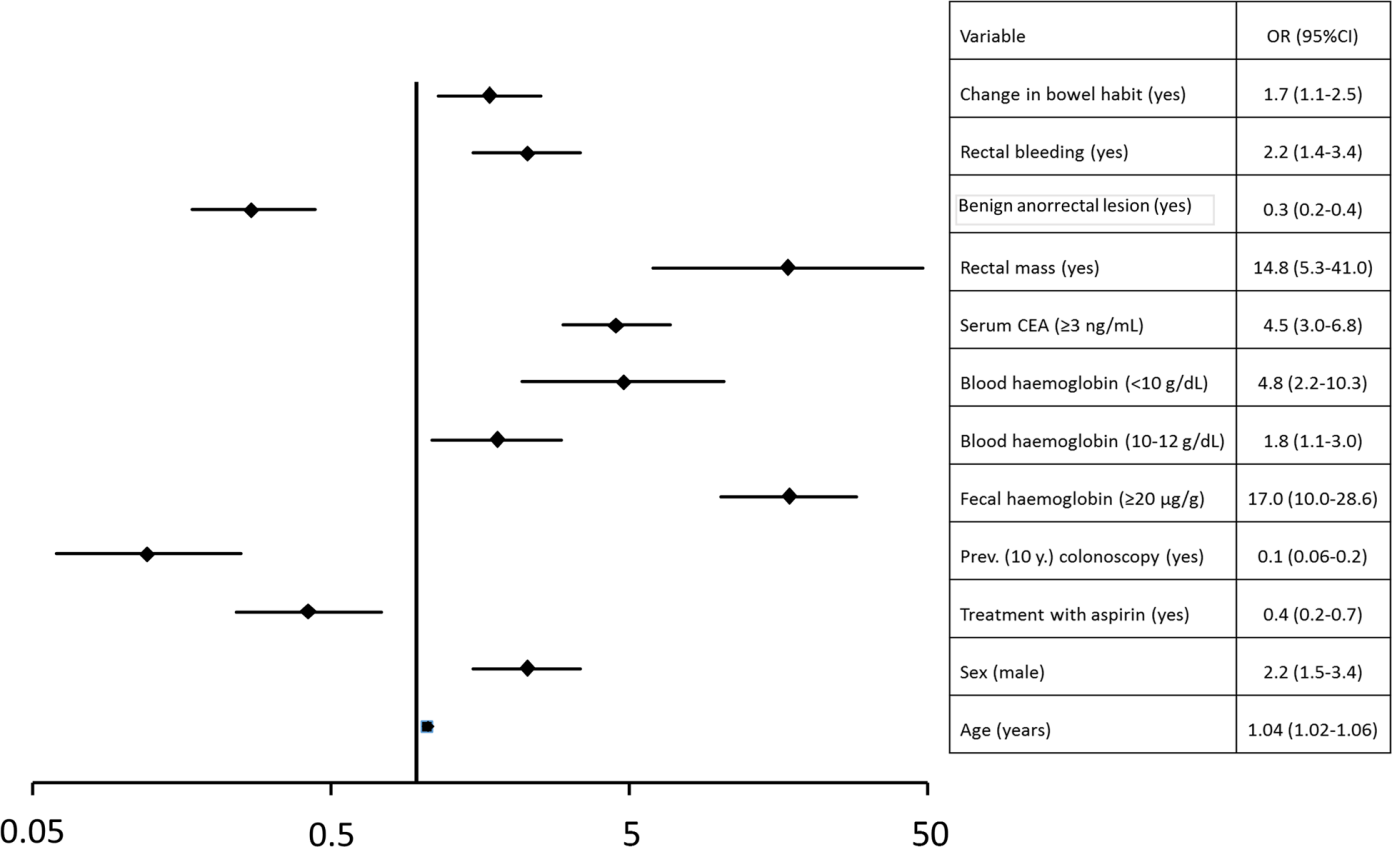
Variables included in the COLONPREDICT model. The relationship with colorectal cancer risk in the multivariate logistic regression model is expressed as the odds ratio (*OR*) and its 95 % confidence interval (*CI*). *CEA* carcinoembryonic antigen; *Prev* previous

In Table 1 we show the results from the initial analyses performed to determine which variables were associated with the risk of detecting CRC. Several variables – age, sex, rectal bleeding, primary healthcare referral, change in bowel habit, symptoms lasting 1–12 months, rectal mass and laboratory results – were associated with an increased risk of CRC detection. On the other hand, the presence of abdominal or anal pain, the detection of benign anorectal lesions, a previous colonoscopy or a family history of CRC reduced the risk of CRC detection on colonoscopy. Age had a normal distribution and a linear relationship with the risk of CRC. In contrast, we transformed the rest of the continuous variables into categorical variables before introducing them into the multivariate logistic regression. We introduced the variables on account of their statistical relationship and their clinical relevance. Our final model (Fig. 2) consisted of 11 variables. The mathematical formula to calculate the COLONPREDICT score is as follows: 0.789 × rectal bleeding + 0.536 × change in bowel habit + 2.694 × rectal mass − 1.283 × benign anorectal lesions + 2.831 × f-Hb ≥20 μg/g of faeces + 1.561 × b-Hb <10 g/dL + 0.588 × b-Hb 10–12 g/dL + 1.511 × CEA ≥3 ng/mL + 0.040 × age (years) + 0.813 × sex (male) − 2.073 × previous colonoscopy (last 10 years) − 0.849 × continuous treatment with aspirin. The intercept of the logistic regression that the COLONPREDICT Score is based on is −7.807. As an example, a 70-year-old man with rectal bleeding, no change in bowel habit, haemorrhoids with no rectal mass on anorectal examination, no previous colonoscopy, continuous treatment with aspirin, serum CEA = 0.2 ng/mL, b-Hb =14 g/dL and a f-Hb of 50 μg/g of faeces would have a COLONPREDICT score of 5.1.

The R2 of our prediction model was 0.55 and the AUC was 0.92 (95 % CI 0.91–0.93). The AIC and BIC were 1213 and 1220. Previously we performed several prediction models with different combinations of variables. We show some of the prediction models evaluated as an example: FIT and rectal mass (AUC 0.85, 95 % CI 0.80–0.85); FIT, CEA, blood haemoglobin and rectal mass (AUC 0.88, 95 % CI 0.86–0.9); and FIT, age, sex, CEA, blood haemoglobin, rectal mass and previous colonoscopy (AUC 0.90, 95 % CI 0.88–0.92). All of them had a significantly inferior discriminatory ability when compared with the final COLONPREDICT model. Finally, a prediction model with the same variables as the COLONPREDICT score but with f-Hb introduced in four strata (undetectable, between 0 and 20 μg Hb/g faeces, between 20 and 200 μg Hb/g faeces, and at least 200 μg Hb/g faeces) had the same discriminatory ability as the final model (AUC 0.92, 95 % CI 0.91–0.94).

### Diagnostic accuracy of the model

**Fig. 3:**
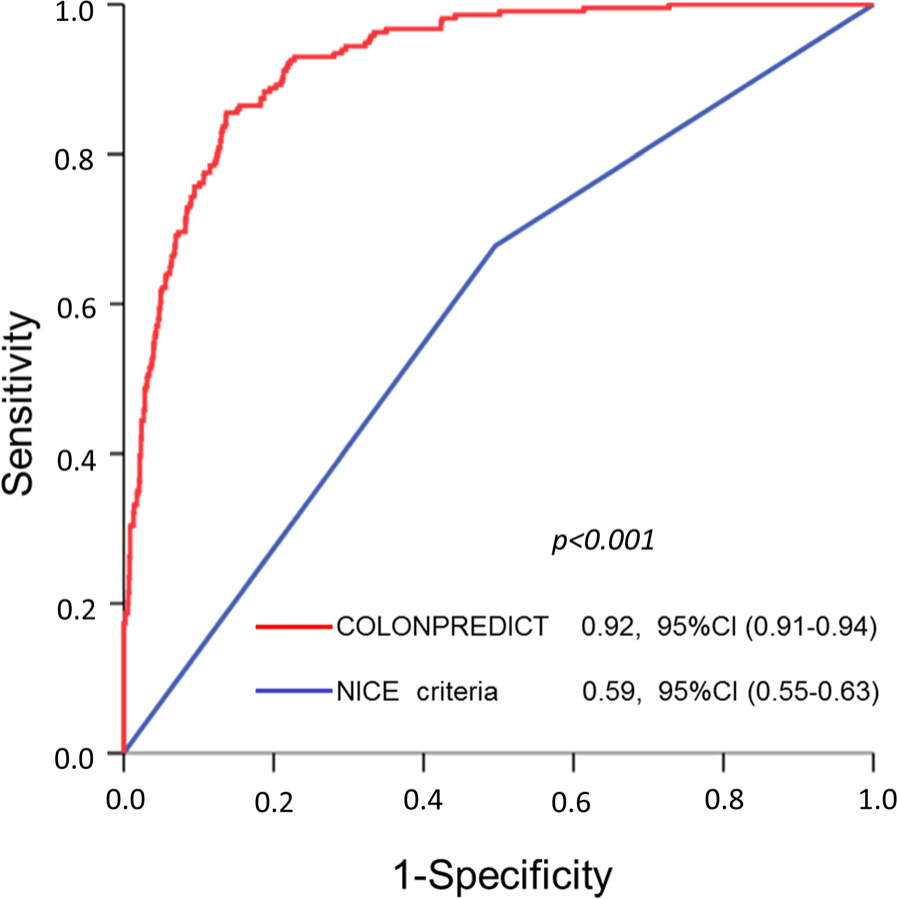
Receiver-operating characteristic (ROC) curve of the COLONPREDICT model and the National Institute for Health and Care Excellence (*NICE*) criteria for colorectal cancer detection in the derivation cohort. The area under the curve (AUC) of the ROC curves are compared with the Chi-square homogeneity area test. *CI* confidence interval

We compared the discriminatory ability of our prediction model with the NICE referral criteria. Overall, the AUC of the COLONPREDICT score was significantly higher than the NICE referral criteria (0.59, 95 % CI 0.55–0.63; *p* < 0.001), as shown in Fig. 3. The example thresholds of the b-coefficient of our prediction model with 90 % and 99 % sensitivity were 5.6 and 3.5, respectively. When comparing the sensitivity and the specificity with the NICE referral criteria, the COLONPREDICT score had higher sensitivity at both thresholds. In contrast, the COLONPREDICT score was less specific than the NICE referral criteria at the 3.5 threshold. The diagnostic accuracy analysis for CRC detection of the NICE referral criteria and the COLONPREDICT score is shown in Table 2. At the example threshold with 50 % sensitivity, the sensitivity, specificity, PPV, negative predictive value (NPV) and number of positives were 53.1 % (46.1–59.9), 96.5 (95.3–97.4), 71.1 % (63.3–77.8), 92.7 % (91.1–94.0) and 10.4 %. In the same way, at the example threshold with 90 % specificity, the sensitivity, specificity, PPV, NPV and number of positives were 77.5 % (71.1–82.8), 89.3 % (87.4–90.9), 53.9 % (48.2–59.6), 96.1 % (94.8–97.1) and 20.0 %

**Table 2:** Diagnostic accuracy of the National Institute for Health and Care Excellence referral criteria and its comparison with the COLONPREDICT score at the thresholds with 90 % (5.6) and 99 % (3.5) sensitivity for colorectal cancer detection in the derivation cohort

	NICE referral criteria	COLONPREDICT score ≥5.6	COLONPREDICT score ≥3.5
Number positives	52.2 %	30.9 %	60.5 %
Sensitivity^a^	68.2 % (61.5–74.3)	90.1 % (85.1–93.6)	99.5 % (97.0–100.0)
Significance^b^		<0.001	<0.001
Specificity^a^	50.3 % (47.6–53.0)	78.7 % (76.4–80.9)	45.8 % (43.1–48.2)
Significance^c^		<0.001	<0.001
Positive predictive value^a^	17.8 % (15.3–20.6)	40.7 % (36.2–45.3)	22.9 % (20.3–25.8)
Negative predictive value^a^	91.0 % (89–93)	98.0 % (96.9–98.7)	99.8 % (98.9–100.0)
Positive likelihood ratio^d^	1.4 (1.2–1.5)	4.2 (3.8–4.7)	1.8 (1.7–1.9)
Negative likelihood ratio^d^	0.6 (0.5–0.8)	0.1 (0.08–0.2)	0.01 (0.0–0.07)
Diagnostic odds ratio^d^	2.2 (1.6–2.9)	33.8 (21.1–54.0)	179 (25–1280)

^a^Values are expressed as the percentage and its 95 % confidence interval

^b^Significance of the sensitivity differences when compared with the NICE referral criteria in the McNemar’s test. Differences with *p* < 0.05 are considered statistically significant

^c^Significance of the specificity differences when compared with the NICE referral criteria in the McNemar’s test. Differences with *p* < 0.05 are considered statistically significant

^d^Values are expressed as the absolute number and its 95 % confidence interval

*NICE* National Institute for Health and Care Excellence

We also analysed the discriminatory ability of the COLONPREDICT score for AN and SCL detection in symptomatic patients. The AUC of the model was 0.83 (0.80–0.85) and 0.82 (0.80–0.84), respectively. The analysis of the sensitivity and specificity at the two example thresholds is shown in Table 3. According to these thresholds, we divided our derivation cohort into three risk groups: high, intermediate and low. We show the diagnostic yield of this classification for CRC, AN and SCL detection in Table 4. In sum, while the number needed to endoscopy to detect a CRC or a SCL was 603 and 11.8 in the low-risk group, the number needed to endoscopy to detect a CRC or a SCL in the high-risk group was 2.5 and 1.6, respectively. The odds ratio (OR) in the high-risk group for CRC detection was 17 (95 % CI 10.5–27) when compared with the intermediate-risk group and 413 (95 % CI 57.5–2961) when compared with the low-risk group. In the same way, patients in the high-risk group had more risk than intermediate- (OR 4.9, 95 % CI 3.7–6.5) and low-risk groups (OR 17.2, 95 % CI 12.3–24.3) for SCL detection.

**Table 3:** Sensitivity and specificity for colorectal cancer and significant colonic lesion of the prediction model at the thresholds with 90 % (5.6) and 99 % (3.5) sensitivity for colorectal cancer detection in the derivation and validation cohorts

COLONPREDICT score		≥5.6		≥3.5	
		Sensitivity^a^	Specificity^a^	Sensitivity^a^	Specificity^a^
CRC	Derivation	90.1 % (85.1–93.6)	78.7 % (76.4–80.9)	99.5 % (97.0–100.0)	45.8 % (43.1–48.2)
	Validation	87.1 % (79.9–92.1)	79.3 % (76.9–81.4)	100 % (96.0–100.0)	46.8 % (44.0–49.6)
	p^b^	0.4	0.7	1	0.6
AN^c^	Derivation	66.7 % (61.8–71.2)	82.3 % (79.9–84.4)	89.5 % (86.1–92.2)	50.1 % (47.2–53.1)
	Validation	66.0 % (60.3–71.3)	83.5 % (81.2–85.7)	88.2 % (83.9–91.5)	50.7 % (47.7–53.7)
	p^b^	0.8	0.4	0.6	0.8
SCL^d^	Derivation	64.2 % (59.5–68.5)	83.1 % (80.7–85.2)	88.7 % (85.3–91.4)	51.3 % (48.3–54.3)
	Validation	59.2 % (53.9–64.3)	84.2 % (81.8–86.3)	84.7 % (80.5–88.2)	51.8 % (48.7–54.9)
	p^b^	0.1	0.5	0.09	0.8

^a^Values are expressed as the percentage and its 95 % confidence interval

^b^Significance of the sensitivity and specificity differences between both cohorts in the Chi-square test. Differences with *p* < 0.05 are considered statistically significant

^c^Colorectal cancer, advanced adenoma (≥10 mm, villous histology, high-grade dysplasia)

^d^Colorectal cancer, advanced adenoma (≥10 mm, villous histology, high-grade dysplasia), polyposis (>10 polyps of any histology), colitis (any aetiology), polyps ≥10 mm, complicated diverticular disease, colonic ulcer and/or bleeding angiodysplasia

*AN* advanced neoplasia, *CRC* colorectal cancer, *SCL* significant colonic lesion

**Table 4:** Diagnostic yield for colorectal cancer and significant colonic lesion detection according to the COLONPREDICT classification in the derivation cohort

		Low risk^a^	Intermediate risk^b^	High risk^c^
Number of patients (%)		39.5	29.6	30.9
Colorectal cancer	PPV (%)^d^	0.2 (0.0– 1.1)	4.4 (2.8–6.8)	40.7 (36.7–45.9)
	NNE (95 % CI)	603 (91–10,000)	22.6 (14.7– 35.7)	2.5 (2.2– 2.7)
	OR (95 % CI)	1.0	27.8 (3.7–208)	413 (57.5–2961)
Advanced neoplasia^e^	PPV (%)^d^	7.1 (5.3– 9.6)	20.8 (17.2– 24.8)	58.1 (53.4– 62.5)
	NNE (95 % CI)	14.1 (10.4– 18.9)	4.8 (4.0– 5.8)	1.7 (1.6– 1.9)
	OR (95 % CI)	1.0	3.4 (2.3–5.0)	18.0 (12.6–25.8)
Significant colonic lesion^f^	PPV (%)^d^	8.5 (6.4– 10.0)	24.5 (20.7–28.8)	61.4 (56.9–65.8)
	NNE (95 % CI)	11.8 (10.0–15.6)	4.1 (3.5– 4.8)	1.6 (1.5– 1.7)
	OR (95 % CI)	1.0	3.5 (2.5–5)	17.2 (12.3–24.3)

^a^Low-risk cohort: COLONPREDICT score <3.5

^b^Intermediate-risk cohort: COLONPREDICT score ≥3.5 and <5.6

^c^High-risk cohort: COLONPREDICT score ≥5.6

^d^Values are expressed as the percentage and its 95 % confidence interval

^e^Colorectal cancer, advanced adenoma (≥10 mm, villous histology, high-grade dysplasia)

^f^Colorectal cancer, advanced adenoma (≥10 mm, villous histology, high-grade dysplasia), polyposis (>10 polyps of any histology), colitis (any aetiology), polyps ≥10 mm, complicated diverticular disease, colonic ulcer and/or bleeding angiodysplasia

*CI* confidence interval, *NNE* number needed to endoscopy, *OR* odds ratio. *PPV* positive predictive value

### Validation of the prediction model

**Fig. 4:**
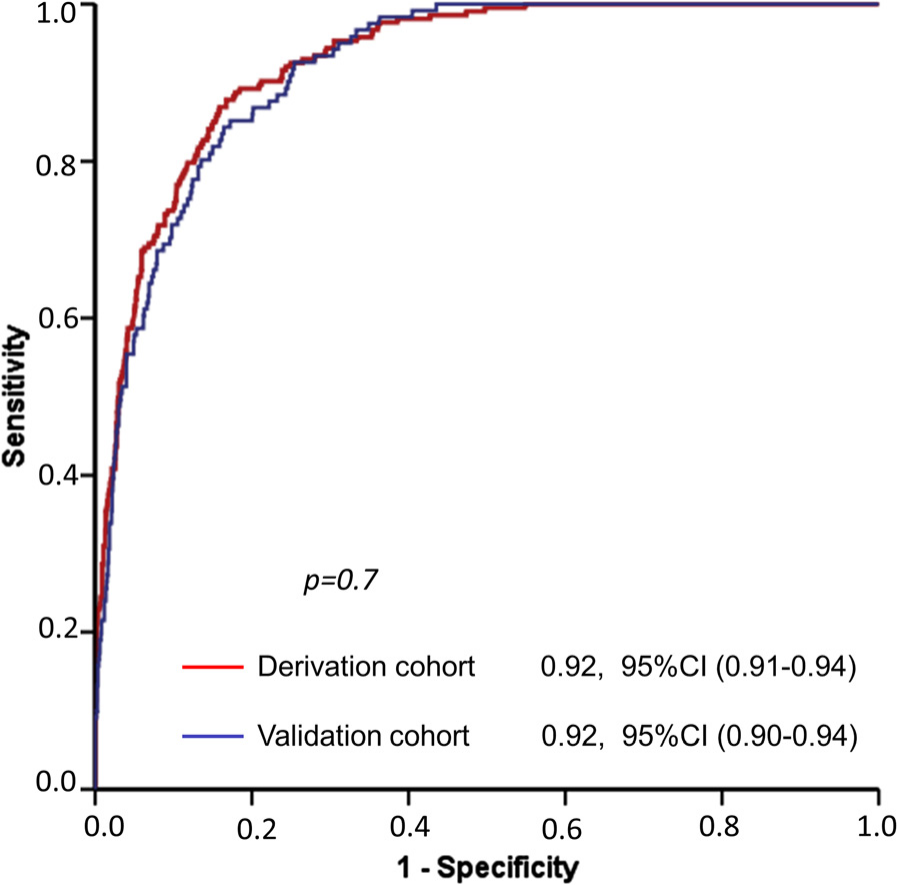
Receiver-operating characteristic (ROC) curve of the COLONPREDICT model for colorectal cancer detection in the derivation and the validation cohort. The area under the curve of the ROC curves are compared with the Chi-square homogeneity area test. *CI* confidence interval

The validation cohort consisted of 1481 patients referred for colonoscopy in 11 hospitals in Spain between March 2014 and March 2015. We show the characteristics of the validation cohort and its comparison with the derivation cohort in Table 5. The validation cohort differed from the derivation cohort with respect to age, primary health care referral, symptoms, treatment with aspirin, benign anorectal lesions, a positive FIT result (≥20 μg Hb/g of faeces), caecal intubation and CRC prevalence. FIT was measured with a qualitative test (HEM-CHECK-2, VEDA-LAB, Alençon Cedex, France) in 22 patients and with a quantitative test in the remaining 1459 patients: 725 with the OC-SENSOR™ (Eiken Chemical Co.), 202 with the OC-Auto 3 Latex™ (Eiken Chemical Co.), 35 with the FOB Gold Test (Sentinel Diagnostics, Milan, Italy), and 497 with Linear i-FOB (Leti, Barcelona, Spain). After using the coefficients to calculate the COLONPREDICT score for each patient in the validation dataset, we compared the discriminatory ability for CRC and SCL detection between both cohorts. We show the results in Fig. 4 and Table 3. The AUC for CRC (0.92, 95 % CI 0.90–0.94; *p* = 0.7), AN (0.82, 95 % CI 0.79–0.85; *p* = 0.5) and SCL (0.78, 95 % CI 0.75–0.81; *p* = 0.05) detection in the validation cohort was similar to the derivation cohort. The −2 log likelihood and the R2 of the model for CRC prediction were 501.1 and 0.49 in the validation dataset, respectively. The Hosmer–Lemeshow test significance was *p* = 0.9 and the calibration plot for CRC detection of the model is shown in Additional file 1: Figure S1. Furthermore, we found no differences in sensitivity of specificity for CRC or SCL detection between both cohorts in the 5.6 and 3.5 thresholds. In the validation cohort, 401 patients (27.1 %) met high-risk group criteria with a 30.3 % (95 % CI 25.8–35.3 %) PPV for CRC detection; 453 (30.6 %) met intermediate-risk group criteria with a 3.5 % (95 % CI 2.1–5.9 %) PPV and 628 patients (42.4 %) met low-risk group criteria with a 0.0 % PPV for CRC detection.

**Table 5:** Comparison of the baseline characteristics of the derivation and validation cohorts

Characteristics	Derivation cohort (*n* = 1572)	Validation cohort (*n* = 1481)	Significance^a^
Age (years)	68 (20–96)	64 (19–101)	<0.001
Sex (male)	810 (51.5 %)	719 (48.5 %)	0.1
Primary healthcare referral (yes)	360 (22.9 %)	570 (38.4 %)	<0.001
Symptoms			
• Change in bowel habit (yes)	899 (57.2 %)	778 (52.6 %)	0.01
• Rectal bleeding (yes)	942 (59.9 %)	758 (51.2 %)	<0.001
Colonoscopy in the last 10 years (yes)	314 (20.0 %)	267 (18.0 %)	0.1
Continuous treatment with aspirin (yes)	224 (14.2 %)	261 (17.6 %)	0.01
Anorectal examination findings			
• Rectal mass (yes)	38 (2.4 %)	27 (1.8 %)	0.2
• Benign anorectal lesion (yes)	646 (41.1 %)	491 (33.2 %)	<0.001
Laboratory results			
• Faecal haemoglobin (≥20 μg/g)	609 (38.7 %)	491 (33.1 %)	0.002
• Blood haemoglobin (<10 g/dL)	75 (4.8 %)	91 (6.1 %)	0.1
• Serum haemoglobin (10–12 g/dL)	251 (16.0 %)	239 (16.1 %)	0.1
• CEA (≥3 ng/mL)	334 (21.2 %)	341 (23.0 %)	0.2
Bowel cleansing (adequate)^b^	1363 (86.7 %)	1274 (86.0 %)	0.7
Complete colonoscopy (yes)	1342 (85.4 %)	1389 (95.0 %)	<0.001
Normal colonoscopy (yes)	855 (38.1 %)	504 (34.0 %)	0.01
Colorectal cancer (yes)	214 (13.6 %)	136 (9.2 %)	<0.001
Significant colonic lesion (yes)^c^	463 (29.5 %)	389 (26.3 %)	0.05
• Advanced adenoma (yes)	251 (16.0 %)	197 (13.3 %)	0.04
• Polyposis (yes)	12 (0.8 %)	11 (0.7 %)	1
• Non adenoma polyp >10 mm (yes)	6 (0.4 %)	26 (1.8 %)	<0.001
• Colitis (yes)	36 (2.3 %)	67 (4.5 %)	0.001

Qualitative variables are expressed as absolute numbers and percentages. Quantitative variables are expressed as median and range

^a^Differences between qualitative variables were analysed with Chi-square test. Differences between quantitative variables were analysed with Student’s *t* test. Differences with *p* < 0.05 are considered statistically significant

^b^Bowel cleansing was adequate if more than 90 % of the mucosa could be evaluated according to the Aronchick scale

^c^Colorectal cancer, advanced adenoma (≥10 mm, villous histology, high-grade dysplasia), polyposis (>10 polyps of any histology), colitis (any aetiology), polyps ≥10 mm, complicated diverticular disease, colonic ulcer and/or bleeding angiodysplasia

*CEA* carcinoembryonic antigen

### Diagnostic accuracy comparison between primary and secondary healthcare referrals

In our post-hoc analysis comparing patients referred from primary and secondary healthcare, we found no significant differences either for CRC (primary 0.91, 95 % CI 0.89–0.94, secondary 0.93, 95 % CI 0.91–0.94; *p* = 0.3), AN (primary 0.83, 95 % CI 0.80–0.87, secondary 0.81, 95 % CI 0.79–0.84; *p* = 0.4) or SCL (primary 0.80, 95 % CI 0.77–0.84, secondary 0.80, 95 % CI 0.77–0.82; *p* = 0.8) detection in the AUC analysis. In addition, apart from a significant difference in specificity for CRC detection at the 90 % sensitivity threshold between both cohorts, we found no differences in the diagnostic accuracy of the COLONPREDICT model as shown in Table 6.

**Table 6:** Sensitivity and specificity for colorectal cancer and significant colonic lesion of the prediction models at the thresholds with 90 % (5.6) and 99 % (3.5) sensitivity for colorectal cancer detection in patients referred for colonoscopy from primary and secondary healthcare

COLONPREDICT score		Colorectal cancer			Advanced neoplasia^a^			Significant colonic lesion^b^		
		Primary	Secondary	*p* ^c^	Primary	Secondary	*p* ^c^	Primary	Secondary	*p* ^c^
≥5.6	Sensitivity^d^	89.0 % (81.6–93.8)	88.9 % (83.9–92.6)	1	70.8 % (64.4–76.5)	64.1 % (59.6–68.4)	0.5	65.3 % (59.3–70.9)	60.2 % (55.9–64.3)	0.1
	Specificity^d^	75.8 % (72.6–78.8)	80.3 % (78.4–82.1)	0.01	80.8 % (77.5–83.7)	83.8 % (81.8–85.6)	1	81.6 % (78.2–84.5)	84.5 % (82.5–86.3)	0.1
≥3.5	Sensitivity^d^	100 % (96.1–100.0)	99.6 % (97.2–100.0)	1	91.4 % (86.8–94.5)	87.8 % (84.4–90.5)	0.1	88.6 % (84.0–92.0)	86.1 % (82.8–88.9)	0.3
	Specificity^d^	44.1 % (40.6–47.7)	47.1 % (44.8–49.4)	0.1	48.8 % (45.0–52.8)	51.0 % (48.4–53.5)	0.6	50.1 % (46.0–54.1)	52.1 % (49.5–54.6)	0.4

^a^Colorectal cancer, advanced adenoma (≥10 mm, villous histology, high-grade dysplasia

^b^Colorectal cancer, advanced adenoma (≥10 mm, villous histology, high-grade dysplasia), polyposis (>10 polyps of any histology), colitis (any aetiology), polyps ≥10 mm, complicated diverticular disease, colonic ulcer and/or bleeding angiodysplasia

^c^Significance of the sensitivity and specificity differences between both cohorts in the Chi-square test. Differences with *p* < 0.05 are considered statistically significant

^d^Values are expressed as the percentage and its 95 % confidence interval

## Discussion and conclusions

### Statement of principal findings

We have developed and externally validated a prediction model for CRC and SCL detection in symptomatic patients referred for colonoscopy. The COLONPREDICT model is based on easily obtainable variables – demographic, laboratory results, symptoms and anorectal examination findings – and is thus applicable both in primary and secondary healthcare. This prediction model is highly accurate, as the calibration plot shows, and allows for differentiation of a high-risk group and, especially, a low-risk group with a probability of CRC detection below 1 %.

### Strengths and weaknesses of the study

We have designed and validated a CRC prediction model on the basis of the hypothesis that symptom-based models had a limited accuracy for CRC detection. We designed our study to compare our prediction model with the NICE referral criteria, the most widely evaluated and implemented criteria for CRC detection. Finally, we were able to validate our prediction model in an external cohort prospectively recruited in several hospitals in Spain, in accordance with the TRIPOP Statement recommendations [23].

However, we believe that the diagnostic accuracy of our prediction model should be externally evaluated in a population with gastrointestinal symptoms attended to in primary care before its use is recommended. Hypothetically, we believe that the diagnostic accuracy of the COLONPREDICT score may increase in CRC low-prevalence populations due to an increase in specificity [10, 12]. Furthermore, although our research has answered the three questions related to a diagnostic test performance identified by Sackett and Haynes before incorporating tests into clinical practice [24], we cannot answer the fourth question: whether patients undergoing the diagnostic test fare better than similar untested patients. Specific research should be carried out in order to evaluate the diagnostic performance in patients with gastrointestinal symptoms evaluated in primary care as well as the efficiency [25].

A secondary outcome of our study is that we have produced the first SCL prediction model in symptomatic patients available in the literature. Furthermore, our score is highly accurate with an AUC of 0.82 and 64.2 and 83.1 % sensitivity at the two thresholds evaluated. We are aware that this score does not exclude the detection of a significant colonic lesions, mainly advanced adenomas. Although advanced adenoma detection is a secondary endpoint of a CRC screening programme, it is not clear that this should be the endpoint in the evaluation of symptomatic patients.

### Strengths and weaknesses in relation to other studies, discussing important differences in results

We have made two main contributions in the design of CRC prediction models. The first one is the inclusion of laboratory findings, mainly FIT, in the prediction model. FIT has recently been evaluated for CRC diagnosis in symptomatic patients and compared with available referral criteria. The available studies show that FIT has a high diagnostic accuracy for CRC detection and our results confirm these findings [8, 15–18]. In fact, the COLONPREDICT score is the first FIT-based CRC prediction model in this setting. Recently, NICE published a new version of the NICE referral criteria for suspected cancer [26]. In this new guideline, they have included offering testing for occult blood in faeces to patients with a PPV below 3 % such as abdominal pain, weight loss, changes in bowel habits or anaemia. Our results suggest that, if faeces are handled appropriately, patients with gastrointestinal symptoms should be evaluated with FIT-based prediction models, even with rectal bleeding. Unfortunately, we could not compare the new NICE referral criteria with our score because the new criteria were published after the study was completed.

Our second main contribution is to determine thresholds based on sensitivity rather than on PPV. The diagnosis of CRC is a balance between the risk of CRC detection and the resources required for the evaluation of patients. Any diagnostic strategy should determine a high-risk group where most of the CRCs are detected and which require a fast-track referral to colonoscopy. But, at the same time, it should also establish a low-risk group where no additional explorations are recommended. In this low-risk group, the probability of missing CRC should be well below 1 %, so that the risk of missing CRC is balanced with the risk of colonoscopy complications, mainly perforation [27]. In this regard, the thresholds with 90 and 99 % sensitivity in our model meet these criteria. In fact, the 99 % sensitivity threshold is consistent with the new NICE guidelines, which aimed to be less specific in order to miss less CRC. Another limitation of the prediction models based on PPVs is that they cannot be transferred to high-prevalence populations. In our opinion, the COLONPREDICT model solves this problem, as we base the referral criteria on sensitivity thresholds. In fact, the number of patients meeting low-risk group criteria would probably increase in low CRC prevalence populations, limiting the resources required for further evaluation [10, 12].

Another important finding of our study is the relevance of the anorectal examination in the evaluation of patients with gastrointestinal symptoms. Although anorectal examination is included within practice guidelines for rectal bleeding evaluation, this information is not included in most of the CRC prediction models available [5, 10, 12, 28]. Moreover, we have confirmed the protective effect of previous colonoscopy and treatment with aspirin in symptomatic patients [29, 30]. However, in the univariate analysis, we did not find a relationship between aspirin and the risk of CRC, which was due to the effect of two confounders: male sex and advanced age. After adjusting for these two variables, aspirin had a protective effect on the risk of detecting a CRC on colonoscopy. Similarly, we found a significant reduction in the risk of detecting a CRC when symptomatic patients had a first-degree relative with CRC in the univariate analysis that was due to the effect of two confounders: female sex and younger age. After adjusting for these two variables, family history had no effect on the risk of detecting a CRC on colonoscopy. Finally, another contribution of our study is the introduction of age as a continuous variable. Available referral criteria use age cut-off points (40, 50 or 60 years) to determine which patients with gastrointestinal symptoms should be evaluated [3, 5, 26], thus hindering the diagnosis of CRC in young patients.

### Unanswered questions and future research

Two main issues need to be answered in the future. As stated before, the diagnostic accuracy and applicability of the COLONPREDICT model in a primary care setting must be addressed in a prospective study. Second, simpler prediction models with similar performance based on laboratory findings must be designed and evaluated in a primary care setting. In this respect, the introduction of new CRC biomarkers may ease the CRC diagnosis process in symptomatic patients.

## Additional file

Additional file 1: Figure S1. Calibration plot of COLONPREDICT model for colorectal cancer detection in the validation cohort. The calibration plot is calculated from the observed and expected proportions within the groups formed by the Hosmer–Lemeshow test. The reference line from the equation is shown. (TIF 739 kb)

## Abbreviations

AIC: Akaike Information Criterion
AN: Advanced neoplasia
AUC: Area under the curve
b-Hb: Blood haemoglobin
BIC: Bayesian Information Criterion
CEA: Carcinoembryonic antigen
CI: Confidence interval
CRC: Colorectal cancer
f-Hb: Faecal haemoglobin
FIT: Faecal immunochemical test
NICE: National Institute for Health and Care Excellence
NNE: Number needed to endoscopy
NPV: Negative predictive value
OR: Odds ratio
PPV: Positive predictive value
ROC: Receiver operating characteristic
SCL: Significant colonic lesion
