# Supplementary figures and images for: Development and external validation of a faecal immunochemical test-based prediction model for colorectal cancer detection in symptomatic patients
Source: BMC Med. 2016 Aug 31;14(1):128. doi: 10.1186/s12916-016-0668-5 (PMC5007726; doi:10.1186/s12916-016-0668-5)

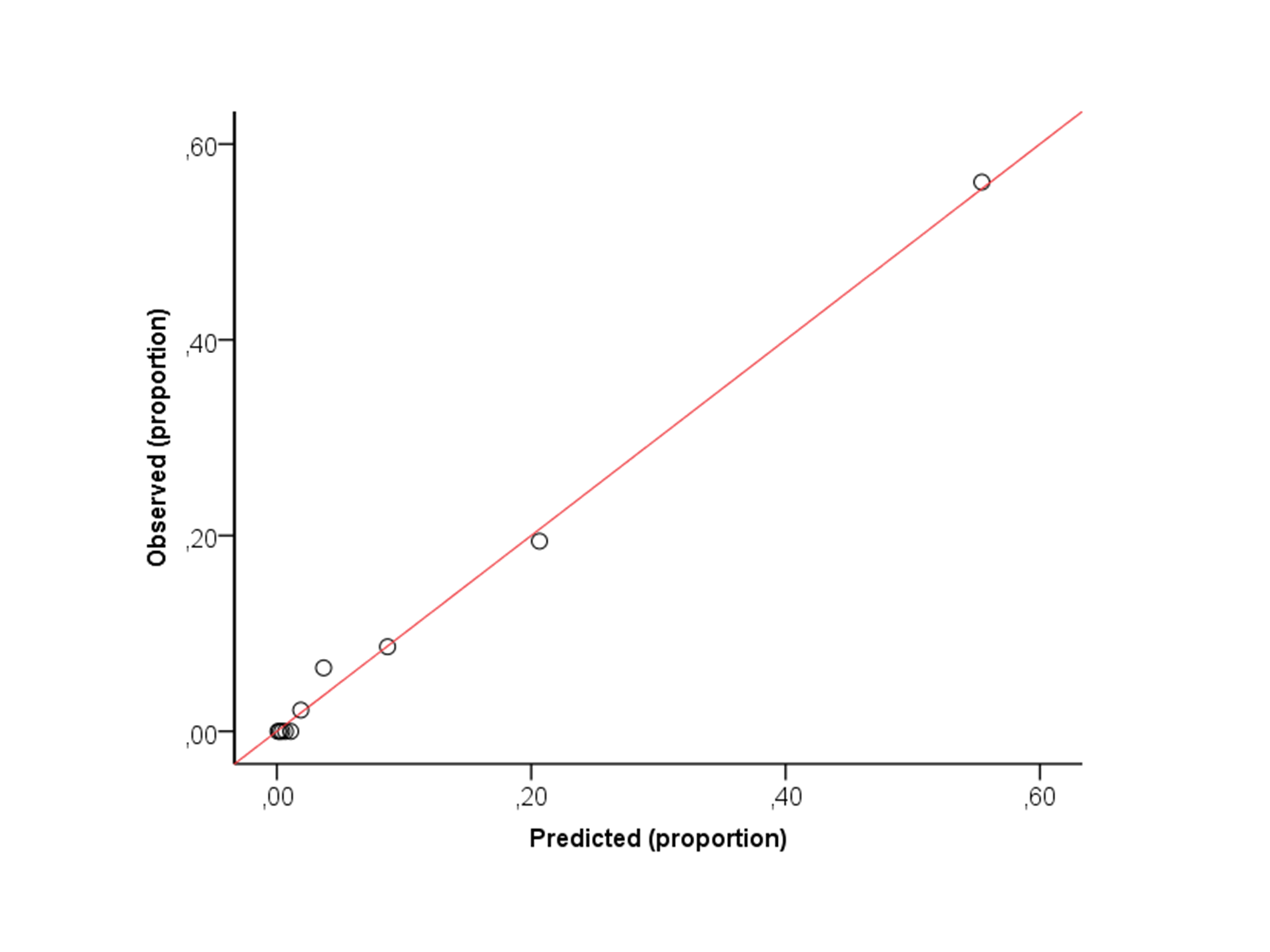

Supplement: Additional file 1: Figure S1. — Calibration plot of COLONPREDICT model for colorectal cancer detection in the validation cohort. The calibration plot is calculated from the observed and expected proportions within the groups formed by the Hosmer–Lemeshow test. The reference line from the equation is shown. (TIF 739 kb) [file 12916_2016_668_MOESM1_ESM.tif]
